# Syntheses and crystal structures of two copper(I)–halide π,σ-coordination compounds based on 2-[(prop-2-en-1-yl)sulfan­yl]pyridine

**DOI:** 10.1107/S2056989021011002

**Published:** 2021-10-29

**Authors:** Yurii Slyvka, Nazariy T. Pokhodylo, Evgeny Goreshnik, Olexii Pavlyuk, Marian Mys’kiv

**Affiliations:** a Department of Inorganic Chemistry, Ivan Franko National University of Lviv, Kyryla i Mefodiya, 6, Lviv, 79005, Ukraine; b Department of Organic Chemistry, Ivan Franko National University of Lviv, Kyryla i Mefodiya, 6, Lviv, 79005, Ukraine; c Department of Inorganic Chemistry and Technology, Jozef Stefan Institute, Jamova 39, SI-1000 Ljubljana, Slovenia

**Keywords:** copper(I), *η* ^2^-inter­action, pyridine-2-thiol, allyl derivative, crystal structure

## Abstract

The title compounds, di-μ-chlorido-bis­({2-[(η-2,3)-(prop-2-en-1-yl)sulfan­yl]pyridine-κ*N*}copper(I)), [Cu_2_Cl_2_(C_8_H_9_NS)_2_], and di-μ-bromido-bis­({2-[(η-2,3)-(prop-2-en-1-yl)sulfan­yl]pyridine-κ*N*}copper(I)), [Cu_2_Br_2_(C_8_H_9_NS)_2_], were obtained by alternating-current electrochemical synthesis starting from an ethano­lic solution of 2-[(prop-2-en-1-yl)sulfan­yl]pyridine (*Psup*) and the copper(II) halide. The isostructural crystals are built up from centrosymmetric [Cu_2_ *Hal*_2_(*Psup*)_2_] dimers, which are formed due to the π,σ-chelating behavior of the organic ligand. In the crystals, the dimers are linked by C—H⋯*Hal* hydrogen bonds as well as by aromatic π–π stacking inter­actions into a three-dimensional network.

## Abstract 2

**Please provide a synopsis (of no more than two sentences) for inclusion in the *Contents* listing of the journal.**

## Chemical context

Cu-containing complexes have been found very promising regarding their catalytic activities in organic syntheses, non-linear optical properties and fluorescent activity (Wang *et al.*, 2005 ▸; Yoshikai & Nakamura, 2012 ▸; Slyvka *et al.*, 2018*a* ▸; Fedorchuk *et al.*, 2020 ▸). Copper complexes also exhibit considerable biochemical activities, ranging from anti­bacterial and anti-inflammatory properties to cytostatic and enzyme inhibitory (Iakovidis *et al.*, 2011 ▸; Tisato *et al.*, 2010 ▸). Some of these compounds have been tested *in vitro* as potential anti­cancer drugs and found to be effective against A549 adenocarcinoma cells that are resistant to the widely used anti­cancer drug cisplatin (Marzano *et al.*, 2006 ▸). It is worth noting that copper is an essential trace element with vital roles in many metalloenzymes participating in intra­cellular processes under normal and pathological conditions (Iakovidis *et al.*, 2011 ▸).

Over the last two decades, increased inter­est has also been devoted to the crystal engineering of copper(I)–olefin complexes with allyl derivatives of heterocyclic compounds (Goreshnik *et al.*, 2011 ▸; Slyvka *et al.*, 2013 ▸; Hordiichuk *et al.*, 2019 ▸). The presence of a C=C olefin bond in a substituent attached to the heterocyclic ring may serve as a key feature for the selective coordination of transition-metal ions due to metal–olefin π-bonding (Rourke, 2006 ▸; Slyvka *et al.*, 2013 ▸; Kowalska *et al.*, 2021 ▸). Allyl derivatives of some heterocyclic compounds were found to be suitable for the preparation of π-coordination compounds with Cu^I^ salts that are unknown (or less stable) in the free state. For instance, the first examples of Cu(C_6_H_5_SO_3_), Cu(*p*-CH_3_C_6_H_4_SO_3_) or CuHSO_4_ π-complexes as well as the direct Cu^I^⋯F(SiF_6_^2–^) inter­action have been observed in copper(I) π-compounds with allyl derivatives of triazole and thia­diazole (Goreshnik *et al.*, 2016 ▸; Ardan *et al.*, 2017 ▸; Slyvka *et al.*, 2018 **b* ▸;* Fedorchuk *et al.*, 2020 ▸). *N*-Allyl derivatives of pyridine were found to be suitable ligands for the crystal engineering of Cu^I^ coordination compounds with inorganic fragments of different complexibility and related to the *pK*_a_ values of the initial pyridine bases (Goreshnik *et al.*, 2003 ▸; Pavlyuk *et al.*, 2005 ▸). Taking into account the fact that allyl­sulfanyl derivatives of pyridine have not been investigated for their coordination behavior regarding copper(I), in this work we present the synthesis and structural characterization of two novel copper(I) halide π-coordination compounds [Cu_2_Cl_2_(*Psup*)_2_] (**I**) & [Cu_2_Br_2_(*Psup*)_2_] (**II**) with 2-[(prop-2-en-1-yl)sulfan­yl]pyridine (*Psup*), C_8_H_9_NS. 
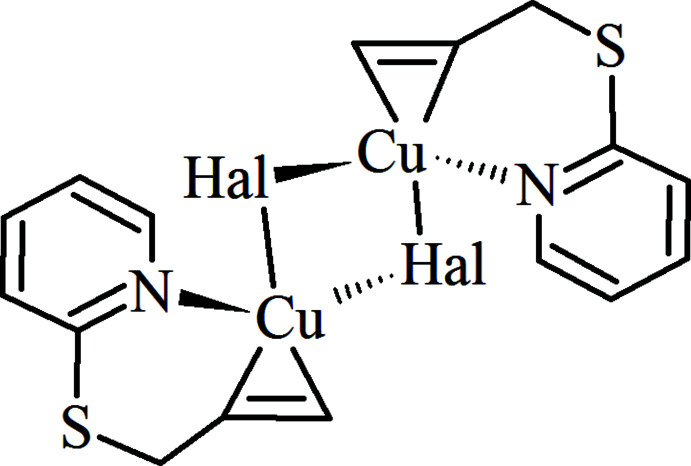


## Structural commentary

**Figure 1:**
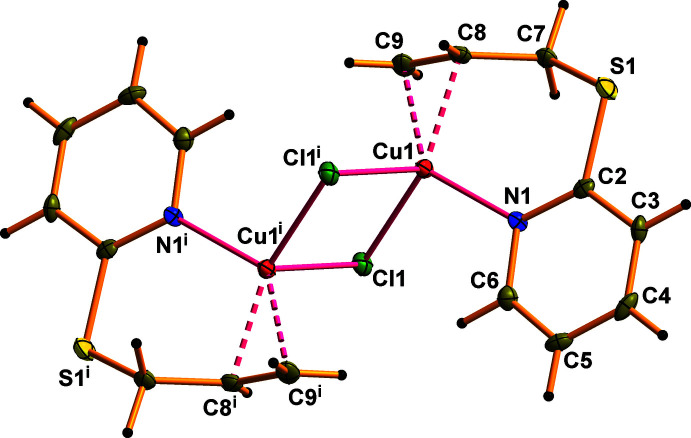
The mol­ecular structure of **I** with displacement ellipsoids drawn at the 50% probability level. Symmetry code: (i) −*x* + 1, −*y* + 1, −*z* + 1.

The title compounds are isostructural and crystallize in the centrosymmetric space group *P*2_1_/*c* with one *Psup* organic mol­ecule, one copper(I) ion and one halide ion in the asymmetric unit. As shown in Figs. 1 ▸ and 2 ▸, these structures are constructed from centrosymmetric [Cu_2_ *Hal*_2_(*Psup*)_2_] [*Hal* = Cl (**I**) or Br (**II**)] dimers, which are formed due to the chelating behavior of the organic ligand. A close to trigonal–pyramidal coordination environment of the Cu^I^ cation includes the *η* ^2^ allylic C=C bond, the pyridine N atom and a *Hal*1 ion in the basal plane (Tables 1 ▸ and 2 ▸). The apical position of the Cu^I^ polyhedron is occupied by the *Hal*1^i^ [symmetry code: (i) −*x* + 1, −*y* + 1, −*z* + 1) ion at 2.6186 (9) Å in **I** and at 2.7113 (6) Å in **II**. The corresponding four-coordinate geometry indices *τ*_4_ (Yang *et al.*, 2007 ▸) are 0.81 (**I**) and 0.83 (**II**). For comparison, in the structures of previously studied CuCl and CuBr π,σ-complexes with allyl­acetoneoxime, the Cu—*Hal*_ap_ distances are slightly higher at 2.719 (5) and 2.778 (4) Å (Filinchuk *et al.*, 1998 ▸).

Being π-connected to the metal center, the C8=C9 bond of the ligand is elongated due to back-donation from an occupied 3*d* metal orbital to a low-lying empty π*-orbital of the olefin to 1.364 (4) Å (**I**) and to 1.354 (6) Å (**II**) in comparison with an uncoordinated allylic C=C bond (Slyvka *et al.*, 2021 ▸). The allyl­sulfanyl group in (**I**) and (**II**) has synclinal conformation relative to the S1—C7 bond and an anti­periplanar conformation relative to the C7—C8 bond [the corresponding torsion angles C2—S1—C7—C8 and S1—C7—C8—C9 are 68.1 (3) and −152.1 (3)°, respectively, in **I** and 68.3 (3) and −151.7 (3)°(**II**)].

## Supra­molecular features

**Figure 3:**
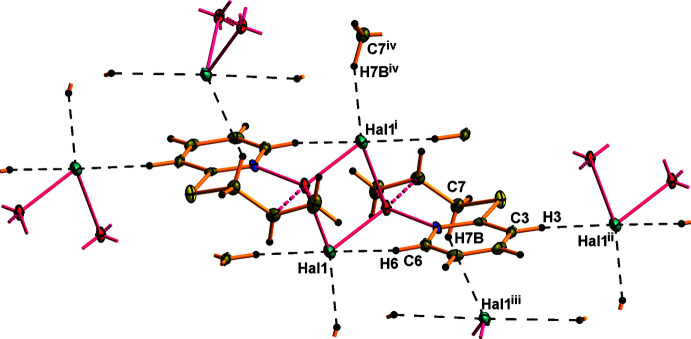
Fragment of the extended structure of **I** with hydrogen bonds shown as dashed lines. Symmetry codes: (i) −*x* + 1, −*y* + 1, −*z* + 1; (ii) *x* + 1, *y*, *z*; (iii) −*x* + 1, *y* + 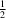 , −*z* + 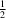 ; (iv) *x*, −*y* + 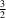 , −*z* + 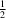 . The packing for **II** is essentially identical.

**Figure 4:**
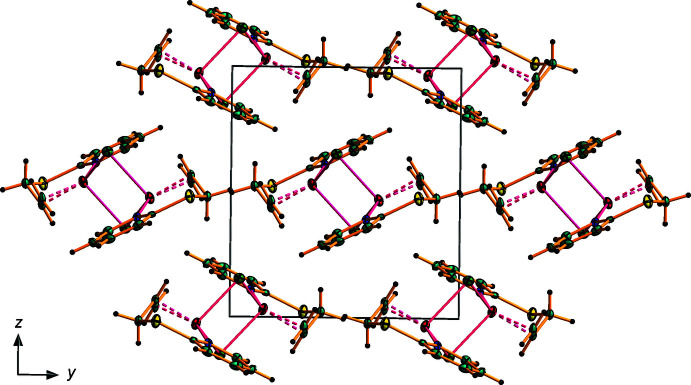
A view along the *a-*axis direction of the crystal packing of **I**.

As shown in Fig. 3 ▸ and listed in Tables 3 ▸ and 4 ▸, the crystal structures of (**I**) and (**II**) features several weak inter­molecular inter­actions. The hydrogen atom H6 of the pyridine ring participates in an intra­molecular C—H⋯*Hal* bond with the *Hal* ion of the inorganic subunit. The other hydrogen atom H6 of the pyridine ring and the methyl­ene hydrogen atom H7*B* of the allyl­sulfanyl substituent are involved in inter­molecular C—H⋯*Hal* bonding, linking the dimeric moieties into a three-dimensional network. The pyridine rings of adjacent dimers are also involved in face-to-face π–π stacking inter­actions with a centroid–centroid separation of 3.680 (4) Å in **I** and 3.693 (4) Å in **II**. The unit-cell packing for (**I**) is shown in Fig. 4 ▸.

## Database survey

The most closest related compounds to the title compounds, containing a similar {Cu_2_ *Hal*_2_} dimer in which a π,σ-chelating ligand is bound to copper(I) are: di-*μ*-chloro­bis­[(1-allyl-3,5-di­methyl­pyrazole)­copper(I)] (III) [Cambridge Structural Database (Version 2021.1; Groom *et al.*, 2016 ▸) refcode ALMPCU; Fukushima *et al.*, 1976 ▸], bis­(*μ*_2_-chloro)-bis­(*η* ^2^-allyl­acetoneoxime-*N*)dicopper(I) (IV) (GOKYAG; Filinchuk *et al.*, 1998 ▸), bis­(*μ*_2_-bromo)-bis­(*η* ^2^-allyl­acetoneoxime-*N*)dicopper(I) (V) (GOKYEK; Filinchuk *et al.*, 1998 ▸), bis­[(*μ*_2_-bromo)(*η* ^2^-2-(allyl­thio)­benzimidazole-*N*)copper(I)] (VI) (WUCRAN; Goreshnik *et al.*, 2002 ▸) and bis­{(*μ*_2_-iodo)[(*η* ^2^-all­yl)(2-pyrid­yl)di­methyl­silane]copper} (VII) (XAZGIP; Kamei *et al.*, 2005 ▸).

Compounds (III) and (VII) crystallize in the triclinic crystal system in space group *P* 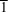 . Compounds (IV), (V) and (VI) crystallize in the monoclinic crystal system in space group *P*2_1_/*c* (settings *P*2_1_/*a*, *P*2_1_/*c* and *P*2_1_/*n*, respectively). Structures (III), (IV), (V) and (VI) are built up from centrosymmetric [Cu_2_ *Hal*_2_(Ligand)_2_] dimers. In the compounds bis­[(*μ*_2_-chloro)­chloro­(*η* ^2^-1-allyl-2-amino­pyridinium)copper(I)] (XIII) (BEBFOE) and bis­[(*μ*_2_-chloro)­bromo­(*η* ^2^-1-allyl-2-amino­pyridinium)copper(I)] (IX) (BEBGAR; Goreshnik *et al.*, 2003 ▸), the 1-allyl-2-amino­pyridinium cation acts as a monodentate π-ligand, being attached to the centrosymmetic anionic {Cu_2_Hal_4_}^2−^ part through the allylic C=C bond. An analogous monodentate 1-allyl­pyridinium cation in the structure of *catena*-[bis­(*μ*_3_-chloro)­bis­(*μ*_2_-chloro)­bis­(*η* ^2^-1-allyl­pyridinium)di­chloro­tetra­copper(I)] (X) (YAPQIQ; Pavlyuk *et al.*, 2005 ▸) forces the realization of an infinite {Cu_4_Cl_4_}_*n*_ inorganic chain.

## Synthesis and crystallization

Crystals of the title compounds were obtained under conditions of alternating-current electrochemical synthesis (Slyvka *et al.*, 2018*a* ▸) starting from an ethano­lic solution of 2-[(prop-2-en-1-yl)sulfan­yl]pyridine (*Psup*) and the copper(II) halide. For this, a solution of *Psup* (1.5 mmol, 0.227 g) in 2.0 ml of 96% ethanol was added to a solution of CuCl_2_·2H_2_O (1.6 mmol, 0.273 g) or CuBr_2_ (1.6 mmol, 0.357 g) in 3.0 ml of 96% ethanol. The mixture was carefully stirred and then was placed into a small 5.5 ml test tube. A copper wire was wrapped into a spiral of 1 cm diameter. A straight copper wire was placed inside the spiral. These copper electrodes were inserted in a cork and immersed in the aforementioned mixture. The mixture was subjected to alternating current reduction (frequency 50 Hz, voltage 0.45 V) and after 3–4 days, good-quality slightly yellowish crystals of the title compounds appeared on the copper wire electrodes. Compound **I**: yield 12%, m.p. 413 K; compound **II**: yield 8%, m.p. 407 K.

## Refinement

Crystal data, data collection and structure refinement details are summarized in Table 5 ▸. All H atoms were positioned geometrically with C—H = 0.95–0.99 Å and refined as riding atoms. The constraint *U*_iso_(H) = 1.2*U*_eq_(C) was applied in all cases.

## Supplementary Material

Crystal structure: contains datablock(s) I, II, publication_text. DOI: 10.1107/S2056989021011002/hb7993sup1.cif

Structure factors: contains datablock(s) I. DOI: 10.1107/S2056989021011002/hb7993Isup2.hkl

Structure factors: contains datablock(s) II. DOI: 10.1107/S2056989021011002/hb7993IIsup3.hkl

CCDC references: 2116969, 2116968

Additional supporting information:  crystallographic information; 3D view; checkCIF report

## Figures and Tables

**Figure 2:**
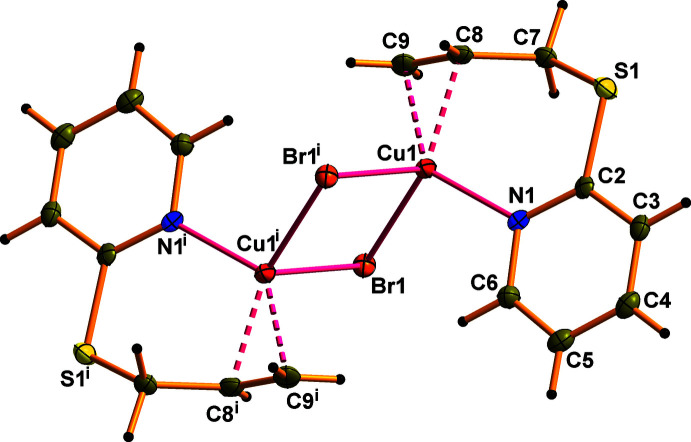
The mol­ecular structure of **II** with displacement ellipsoids drawn at the 50% probability level. Symmetry code: (i) −*x* + 1, −*y* + 1, −*z* + 1.

**Table 1:** Selected bond lengths (Å) for **I** [Chem scheme1]

Cu1—Cl1	2.2691 (9)	Cu1—C8	2.037 (3)
Cu1—Cl1^i^	2.6186 (9)	Cu1—C9	2.052 (3)
Cu1—N1	2.026 (2)		

Symmetry code: (i) -x+1, -y+1, -z+1.

**Table 2:** Selected bond lengths (Å) for **II** [Chem scheme1]

Cu1—Br1	2.4097 (6)	Cu1—C8	2.048 (4)
Cu1—Br1^i^	2.7113 (6)	Cu1—C9	2.065 (4)
Cu1—N1	2.025 (3)		

Symmetry code: (i) -x+1, -y+1, -z+1.

**Table 3:** Hydrogen-bond geometry (Å, °) for **I** [Chem scheme1]

*D*—H⋯*A*	*D*—H	H⋯*A*	*D*⋯*A*	*D*—H⋯*A*
C3—H3⋯Cl1^ii^	0.95	2.91	3.581 (3)	129
C6—H6⋯Cl1	0.95	2.80	3.447 (3)	126
C7—H7*B*⋯Cl1^iii^	0.99	2.89	3.676 (3)	137

Symmetry codes: (ii) x+1, y, z; (iii) -x+1, y+{\script{1\over 2}}, -z+{\script{1\over 2}}.

**Table 4:** Hydrogen-bond geometry (Å, °) for **II** [Chem scheme1]

*D*—H⋯*A*	*D*—H	H⋯*A*	*D*⋯*A*	*D*—H⋯*A*
C3—H3⋯Br1^ii^	0.95	3.02	3.696 (4)	129
C6—H6⋯Br1	0.95	2.94	3.576 (4)	126
C7—H7*B*⋯Br1^iii^	0.99	2.94	3.744 (4)	139

Symmetry codes: (ii) x+1, y, z; (iii) -x+1, y+{\script{1\over 2}}, -z+{\script{1\over 2}}.

**Table 5:** Experimental details

	**I**	**II**
Crystal data		
Chemical formula	[Cu_2_Cl_2_(C_8_H_9_NS)_2_]	[Cu_2_Br_2_(C_8_H_9_NS)_2_]
*M*_r_	500.42	589.34
Crystal system, space group	Monoclinic, *P*2_1_/*c*	Monoclinic, *P*2_1_/*c*
Temperature (K)	150	150
*a*, *b*, *c* (Å)	9.2729 (16), 9.5740 (13), 11.037 (2)	9.5009 (6), 9.6022 (5), 11.0936 (8)
β (°)	108.52 (2)	107.257 (7)
*V* (Å^3^)	929.1 (3)	966.50 (11)
*Z*	2	2
Radiation type	Mo *K*α	Mo *K*α
μ (mm^−1^)	2.80	6.55
Crystal size (mm)	0.33 × 0.28 × 0.19	0.44 × 0.35 × 0.22

Data collection		
Diffractometer	Rigaku New Gemini, Dual, Atlas	Rigaku New Gemini, Dual, Atlas
Absorption correction	Analytical (*CrysAlis PRO*; Rigaku OD, 2021 ▸)	Analytical (*CrysAlis PRO*; Rigaku OD, 2021 ▸)
*T*_min_, *T*_max_	0.546, 0.693	0.191, 0.368
No. of measured, independent and observed [*I* > 2σ(*I*)] reflections	8088, 2161, 1730	6837, 2162, 1854
*R*_int_	0.058	0.044
(sin θ/λ)_max_ (Å^−1^)	0.686	0.682

Refinement		
*R*[*F* ^2^ > 2σ(*F* ^2^)], *wR*(*F* ^2^), *S*	0.036, 0.077, 1.08	0.034, 0.079, 1.08
No. of reflections	2161	2162
No. of parameters	109	109
H-atom treatment	H-atom parameters constrained	H-atom parameters constrained
Δρ_max_, Δρ_min_ (e Å^−3^)	0.51, −0.64	0.82, −0.75

Computer programs: *CrysAlis PRO* (Rigaku OD, 2021 ▸), *SHELXT* (Sheldrick, 2015*a* ▸), *SHELXL* (Sheldrick, 2015*b* ▸) and *OLEX2* (Dolomanov *et al.*, 2009 ▸).

## supplementary crystallographic information

### Di-µ-chlorido-bis({2-[(η-2,3)-(prop-2-en-1-yl)sulfanyl]pyridine-κN}copper(I)) (I) Crystal data

**Table:**

[Cu_2_Cl_2_(C_8_H_9_NS)_2_]	*F*(000) = 504
*M**_r_* = 500.42	*D*_x_ = 1.789 Mg m^−^^3^
Monoclinic, *P*2_1_/*c*	Mo *K*α radiation, λ = 0.71073 Å
*a* = 9.2729 (16) Å	Cell parameters from 3255 reflections
*b* = 9.5740 (13) Å	θ = 3.8–28.9°
*c* = 11.037 (2) Å	µ = 2.80 mm^−^^1^
β = 108.52 (2)°	*T* = 150 K
*V* = 929.1 (3) Å^3^	Irregular, yellowish
*Z* = 2	0.33 × 0.28 × 0.19 mm

### Di-µ-chlorido-bis({2-[(η-2,3)-(prop-2-en-1-yl)sulfanyl]pyridine-κN}copper(I)) (I) Data collection

**Table:**

New Gemini, Dual, Cu at home/near, Atlas diffractometer	1730 reflections with *I* > 2σ(*I*)
Detector resolution: 10.6426 pixels mm^-1^	*R*_int_ = 0.058
ω scans	θ_max_ = 29.2°, θ_min_ = 2.9°
Absorption correction: analytical (CrysalisPro; Rigaku OD, 2021)	*h* = −12→12
*T*_min_ = 0.546, *T*_max_ = 0.693	*k* = −12→11
8088 measured reflections	*l* = −15→15
2161 independent reflections	

### Di-µ-chlorido-bis({2-[(η-2,3)-(prop-2-en-1-yl)sulfanyl]pyridine-κN}copper(I)) (I) Refinement

**Table:**

Refinement on *F*^2^	Primary atom site location: dual
Least-squares matrix: full	Hydrogen site location: inferred from neighbouring sites
*R*[*F*^2^ > 2σ(*F*^2^)] = 0.036	H-atom parameters constrained
*wR*(*F*^2^) = 0.077	*w* = 1/[σ^2^(*F*_o_^2^) + (0.0228*P*)^2^ + 0.6316*P*] where *P* = (*F*_o_^2^ + 2*F*_c_^2^)/3
*S* = 1.08	(Δ/σ)_max_ = 0.001
2161 reflections	Δρ_max_ = 0.51 e Å^−^^3^
109 parameters	Δρ_min_ = −0.64 e Å^−^^3^
0 restraints	

### Di-µ-chlorido-bis({2-[(η-2,3)-(prop-2-en-1-yl)sulfanyl]pyridine-κN}copper(I)) (I) Special details

**Table:**

Geometry. All esds (except the esd in the dihedral angle between two l.s. planes) are estimated using the full covariance matrix. The cell esds are taken into account individually in the estimation of esds in distances, angles and torsion angles; correlations between esds in cell parameters are only used when they are defined by crystal symmetry. An approximate (isotropic) treatment of cell esds is used for estimating esds involving l.s. planes.

### Di-µ-chlorido-bis({2-[(η-2,3)-(prop-2-en-1-yl)sulfanyl]pyridine-κN}copper(I)) (I) Fractional atomic coordinates and isotropic or equivalent isotropic displacement parameters (Å^2^)

**Table:**

	*x*	*y*	*z*	*U*_iso_*/*U*_eq_	
Cu1	0.57740 (4)	0.64590 (4)	0.46958 (4)	0.01225 (12)	
Cl1	0.36070 (8)	0.54060 (8)	0.34752 (7)	0.01340 (18)	
S1	0.91865 (9)	0.82046 (8)	0.47781 (8)	0.01693 (19)	
N1	0.7401 (3)	0.5908 (2)	0.3922 (2)	0.0098 (5)	
C2	0.8718 (3)	0.6558 (3)	0.4035 (3)	0.0113 (6)	
C3	0.9837 (3)	0.5968 (3)	0.3594 (3)	0.0142 (7)	
H3	1.075431	0.645998	0.368256	0.017*	
C4	0.9603 (4)	0.4679 (3)	0.3036 (3)	0.0175 (7)	
H4	1.036450	0.425281	0.275400	0.021*	
C5	0.8239 (4)	0.4003 (3)	0.2888 (3)	0.0158 (7)	
H5	0.803694	0.311139	0.249195	0.019*	
C6	0.7184 (3)	0.4656 (3)	0.3331 (3)	0.0145 (7)	
H6	0.624182	0.419633	0.321361	0.017*	
C7	0.7410 (4)	0.8972 (3)	0.4778 (3)	0.0145 (7)	
H7A	0.759319	0.996020	0.504812	0.017*	
H7B	0.669754	0.896375	0.389294	0.017*	
C8	0.6665 (4)	0.8249 (3)	0.5633 (3)	0.0137 (7)	
H8	0.729407	0.776905	0.636305	0.016*	
C9	0.5133 (4)	0.8248 (3)	0.5412 (3)	0.0198 (8)	
H9A	0.447951	0.872113	0.468765	0.024*	
H9B	0.471792	0.777530	0.598143	0.024*	

### Di-µ-chlorido-bis({2-[(η-2,3)-(prop-2-en-1-yl)sulfanyl]pyridine-κN}copper(I)) (I) Atomic displacement parameters (Å^2^)

**Table:**

	*U*^11^	*U*^22^	*U*^33^	*U*^12^	*U*^13^	*U*^23^
Cu1	0.0103 (2)	0.0106 (2)	0.0177 (2)	−0.00093 (15)	0.00702 (17)	−0.00293 (16)
Cl1	0.0082 (4)	0.0164 (4)	0.0146 (4)	−0.0007 (3)	0.0022 (3)	0.0002 (3)
S1	0.0130 (4)	0.0140 (4)	0.0249 (5)	−0.0034 (3)	0.0076 (4)	−0.0022 (3)
N1	0.0066 (12)	0.0092 (12)	0.0125 (13)	0.0009 (10)	0.0013 (11)	0.0005 (11)
C2	0.0131 (15)	0.0132 (15)	0.0063 (14)	0.0031 (13)	0.0013 (13)	0.0022 (12)
C3	0.0097 (15)	0.0187 (17)	0.0160 (16)	0.0024 (13)	0.0064 (13)	0.0057 (14)
C4	0.0147 (16)	0.0270 (19)	0.0129 (16)	0.0101 (14)	0.0073 (14)	0.0026 (15)
C5	0.0192 (17)	0.0145 (16)	0.0128 (16)	0.0050 (14)	0.0037 (14)	−0.0029 (13)
C6	0.0117 (15)	0.0160 (16)	0.0162 (16)	0.0010 (13)	0.0049 (14)	0.0018 (13)
C7	0.0176 (17)	0.0091 (15)	0.0184 (16)	−0.0004 (13)	0.0079 (14)	−0.0004 (13)
C8	0.0182 (17)	0.0080 (15)	0.0154 (16)	−0.0003 (13)	0.0059 (14)	−0.0017 (13)
C9	0.0245 (19)	0.0091 (16)	0.031 (2)	0.0003 (14)	0.0164 (16)	−0.0026 (14)

### Di-µ-chlorido-bis({2-[(η-2,3)-(prop-2-en-1-yl)sulfanyl]pyridine-κN}copper(I)) (I) Geometric parameters (Å, º)

**Table:**

Cu1—Cl1	2.2691 (9)	C4—H4	0.9500
Cu1—Cl1^i^	2.6186 (9)	C4—C5	1.383 (4)
Cu1—N1	2.026 (2)	C5—H5	0.9500
Cu1—C8	2.037 (3)	C5—C6	1.375 (4)
Cu1—C9	2.052 (3)	C6—H6	0.9500
S1—C2	1.766 (3)	C7—H7A	0.9900
S1—C7	1.804 (3)	C7—H7B	0.9900
N1—C2	1.340 (4)	C7—C8	1.503 (4)
N1—C6	1.349 (4)	C8—H8	0.9500
C2—C3	1.397 (4)	C8—C9	1.364 (4)
C3—H3	0.9500	C9—H9A	0.9500
C3—C4	1.366 (4)	C9—H9B	0.9500
			
Cl1—Cu1—Cl1^i^	95.20 (3)	C4—C5—H5	120.9
N1—Cu1—Cl1^i^	97.91 (7)	C6—C5—C4	118.1 (3)
N1—Cu1—Cl1	105.77 (7)	C6—C5—H5	120.9
N1—Cu1—C8	101.34 (11)	N1—C6—C5	124.2 (3)
N1—Cu1—C9	136.50 (12)	N1—C6—H6	117.9
C8—Cu1—Cl1^i^	103.19 (9)	C5—C6—H6	117.9
C8—Cu1—Cl1	144.63 (9)	S1—C7—H7A	108.7
C8—Cu1—C9	38.96 (12)	S1—C7—H7B	108.7
C9—Cu1—Cl1^i^	106.96 (10)	H7A—C7—H7B	107.6
C9—Cu1—Cl1	106.81 (10)	C8—C7—S1	114.4 (2)
Cu1—Cl1—Cu1^i^	84.80 (3)	C8—C7—H7A	108.7
C2—S1—C7	105.89 (15)	C8—C7—H7B	108.7
C2—N1—Cu1	128.2 (2)	Cu1—C8—H8	93.7
C2—N1—C6	116.7 (3)	C7—C8—Cu1	105.2 (2)
C6—N1—Cu1	114.72 (19)	C7—C8—H8	118.4
N1—C2—S1	122.7 (2)	C9—C8—Cu1	71.15 (18)
N1—C2—C3	122.3 (3)	C9—C8—C7	123.2 (3)
C3—C2—S1	115.0 (2)	C9—C8—H8	118.4
C2—C3—H3	120.2	Cu1—C9—H9A	105.0
C4—C3—C2	119.5 (3)	Cu1—C9—H9B	94.9
C4—C3—H3	120.2	C8—C9—Cu1	69.90 (18)
C3—C4—H4	120.5	C8—C9—H9A	120.0
C3—C4—C5	119.0 (3)	C8—C9—H9B	120.0
C5—C4—H4	120.5	H9A—C9—H9B	120.0
			
Cu1—N1—C2—S1	−7.6 (4)	C2—C3—C4—C5	1.8 (5)
Cu1—N1—C2—C3	171.1 (2)	C3—C4—C5—C6	−1.0 (5)
Cu1—N1—C6—C5	−171.2 (2)	C4—C5—C6—N1	−1.1 (5)
S1—C2—C3—C4	178.2 (2)	C6—N1—C2—S1	179.9 (2)
S1—C7—C8—Cu1	−75.0 (2)	C6—N1—C2—C3	−1.4 (4)
S1—C7—C8—C9	−152.1 (3)	C7—S1—C2—N1	−19.4 (3)
N1—C2—C3—C4	−0.6 (5)	C7—S1—C2—C3	161.8 (2)
C2—S1—C7—C8	68.1 (3)	C7—C8—C9—Cu1	96.2 (3)
C2—N1—C6—C5	2.3 (4)		

Symmetry code: (i) −*x*+1, −*y*+1, −*z*+1.

### Di-µ-chlorido-bis({2-[(η-2,3)-(prop-2-en-1-yl)sulfanyl]pyridine-κN}copper(I)) (I) Hydrogen-bond geometry (Å, º)

**Table:**

*D*—H···*A*	*D*—H	H···*A*	*D*···*A*	*D*—H···*A*
C3—H3···Cl1^ii^	0.95	2.91	3.581 (3)	129
C6—H6···Cl1	0.95	2.80	3.447 (3)	126
C7—H7*B*···Cl1^iii^	0.99	2.89	3.676 (3)	137

Symmetry codes: (ii) *x*+1, *y*, *z*; (iii) −*x*+1, *y*+1/2, −*z*+1/2.

### Di-µ-bromido-bis({2-[(η-2,3)-(prop-2-en-1-yl)sulfanyl]pyridine-κN}copper(I)) (II) Crystal data

**Table:**

[Cu_2_Br_2_(C_8_H_9_NS)_2_]	*F*(000) = 576
*M**_r_* = 589.34	*D*_x_ = 2.025 Mg m^−^^3^
Monoclinic, *P*2_1_/*c*	Mo *K*α radiation, λ = 0.71073 Å
*a* = 9.5009 (6) Å	Cell parameters from 3535 reflections
*b* = 9.6022 (5) Å	θ = 3.1–29.0°
*c* = 11.0936 (8) Å	µ = 6.55 mm^−^^1^
β = 107.257 (7)°	*T* = 150 K
*V* = 966.50 (11) Å^3^	Irregular, yellowish
*Z* = 2	0.44 × 0.35 × 0.22 mm

### Di-µ-bromido-bis({2-[(η-2,3)-(prop-2-en-1-yl)sulfanyl]pyridine-κN}copper(I)) (II) Data collection

**Table:**

New Gemini, Dual, Cu at home/near, Atlas diffractometer	1854 reflections with *I* > 2σ(*I*)
Detector resolution: 10.6426 pixels mm^-1^	*R*_int_ = 0.044
ω scans	θ_max_ = 29.0°, θ_min_ = 2.9°
Absorption correction: analytical (CrysalisPro; Rigaku OD, 2021)	*h* = −12→12
*T*_min_ = 0.191, *T*_max_ = 0.368	*k* = −10→12
6837 measured reflections	*l* = −12→13
2162 independent reflections	

### Di-µ-bromido-bis({2-[(η-2,3)-(prop-2-en-1-yl)sulfanyl]pyridine-κN}copper(I)) (II) Refinement

**Table:**

Refinement on *F*^2^	0 restraints
Least-squares matrix: full	Hydrogen site location: inferred from neighbouring sites
*R*[*F*^2^ > 2σ(*F*^2^)] = 0.034	H-atom parameters constrained
*wR*(*F*^2^) = 0.079	*w* = 1/[σ^2^(*F*_o_^2^) + (0.0328*P*)^2^ + 1.3651*P*] where *P* = (*F*_o_^2^ + 2*F*_c_^2^)/3
*S* = 1.08	(Δ/σ)_max_ = 0.001
2162 reflections	Δρ_max_ = 0.82 e Å^−^^3^
109 parameters	Δρ_min_ = −0.74 e Å^−^^3^

### Di-µ-bromido-bis({2-[(η-2,3)-(prop-2-en-1-yl)sulfanyl]pyridine-κN}copper(I)) (II) Special details

**Table:**

Geometry. All esds (except the esd in the dihedral angle between two l.s. planes) are estimated using the full covariance matrix. The cell esds are taken into account individually in the estimation of esds in distances, angles and torsion angles; correlations between esds in cell parameters are only used when they are defined by crystal symmetry. An approximate (isotropic) treatment of cell esds is used for estimating esds involving l.s. planes.

### Di-µ-bromido-bis({2-[(η-2,3)-(prop-2-en-1-yl)sulfanyl]pyridine-κN}copper(I)) (II) Fractional atomic coordinates and isotropic or equivalent isotropic displacement parameters (Å^2^)

**Table:**

	*x*	*y*	*z*	*U*_iso_*/*U*_eq_	
Br1	0.36045 (4)	0.54960 (3)	0.34080 (3)	0.01538 (12)	
Cu1	0.58645 (5)	0.64663 (4)	0.47542 (4)	0.01478 (13)	
S1	0.91726 (10)	0.82315 (9)	0.48313 (10)	0.0201 (2)	
N1	0.7467 (3)	0.5936 (3)	0.3976 (3)	0.0135 (6)	
C2	0.8735 (4)	0.6601 (3)	0.4079 (3)	0.0127 (7)	
C3	0.9838 (4)	0.6033 (4)	0.3631 (3)	0.0176 (8)	
H3	1.072777	0.652922	0.372109	0.021*	
C4	0.9622 (4)	0.4747 (4)	0.3060 (4)	0.0191 (8)	
H4	1.036808	0.433779	0.276414	0.023*	
C5	0.8301 (4)	0.4056 (4)	0.2920 (3)	0.0186 (8)	
H5	0.811443	0.317584	0.251361	0.022*	
C6	0.7270 (4)	0.4681 (4)	0.3387 (4)	0.0168 (8)	
H6	0.636629	0.420650	0.329185	0.020*	
C7	0.7453 (4)	0.8986 (4)	0.4850 (4)	0.0171 (8)	
H7A	0.762903	0.996856	0.512413	0.021*	
H7B	0.677805	0.898701	0.397736	0.021*	
C8	0.6700 (4)	0.8262 (4)	0.5692 (4)	0.0192 (8)	
H8	0.729668	0.778274	0.641149	0.023*	
C9	0.5222 (5)	0.8253 (4)	0.5484 (4)	0.0247 (9)	
H9A	0.459626	0.872317	0.477213	0.030*	
H9B	0.481069	0.777660	0.605052	0.030*	

### Di-µ-bromido-bis({2-[(η-2,3)-(prop-2-en-1-yl)sulfanyl]pyridine-κN}copper(I)) (II) Atomic displacement parameters (Å^2^)

**Table:**

	*U*^11^	*U*^22^	*U*^33^	*U*^12^	*U*^13^	*U*^23^
Br1	0.01321 (19)	0.0189 (2)	0.0130 (2)	−0.00013 (14)	0.00236 (14)	0.00135 (13)
Cu1	0.0151 (2)	0.0135 (2)	0.0174 (3)	−0.00122 (17)	0.00731 (19)	−0.00381 (16)
S1	0.0185 (5)	0.0159 (5)	0.0268 (5)	−0.0045 (4)	0.0081 (4)	−0.0043 (4)
N1	0.0154 (15)	0.0150 (14)	0.0108 (15)	0.0022 (12)	0.0048 (12)	−0.0002 (11)
C2	0.0152 (18)	0.0151 (17)	0.0073 (17)	0.0006 (14)	0.0024 (14)	0.0040 (13)
C3	0.0165 (19)	0.0237 (19)	0.0136 (19)	−0.0004 (15)	0.0063 (15)	0.0019 (14)
C4	0.0192 (19)	0.023 (2)	0.016 (2)	0.0059 (16)	0.0066 (16)	0.0024 (15)
C5	0.021 (2)	0.0209 (19)	0.0142 (19)	0.0041 (15)	0.0054 (15)	−0.0011 (14)
C6	0.0167 (19)	0.0165 (18)	0.018 (2)	−0.0004 (15)	0.0058 (15)	−0.0007 (14)
C7	0.022 (2)	0.0116 (17)	0.019 (2)	−0.0006 (15)	0.0079 (16)	−0.0002 (14)
C8	0.030 (2)	0.0103 (17)	0.019 (2)	−0.0003 (15)	0.0101 (17)	−0.0027 (14)
C9	0.033 (2)	0.0115 (18)	0.036 (2)	−0.0004 (16)	0.020 (2)	−0.0065 (15)

### Di-µ-bromido-bis({2-[(η-2,3)-(prop-2-en-1-yl)sulfanyl]pyridine-κN}copper(I)) (II) Geometric parameters (Å, º)

**Table:**

Cu1—Br1	2.4097 (6)	C4—H4	0.9500
Cu1—Br1^i^	2.7113 (6)	C4—C5	1.387 (5)
Cu1—N1	2.025 (3)	C5—H5	0.9500
Cu1—C8	2.048 (4)	C5—C6	1.374 (5)
Cu1—C9	2.065 (4)	C6—H6	0.9500
S1—C2	1.765 (4)	C7—H7A	0.9900
S1—C7	1.793 (4)	C7—H7B	0.9900
N1—C2	1.338 (5)	C7—C8	1.505 (5)
N1—C6	1.357 (5)	C8—H8	0.9500
C2—C3	1.397 (5)	C8—C9	1.354 (6)
C3—H3	0.9500	C9—H9A	0.9500
C3—C4	1.375 (5)	C9—H9B	0.9500
			
Cu1—Br1—Cu1^i^	82.521 (18)	C4—C5—H5	120.9
Br1—Cu1—Br1^i^	97.479 (19)	C6—C5—C4	118.1 (4)
N1—Cu1—Br1^i^	98.64 (8)	C6—C5—H5	120.9
N1—Cu1—Br1	106.43 (9)	N1—C6—C5	123.9 (4)
N1—Cu1—C8	101.60 (14)	N1—C6—H6	118.1
N1—Cu1—C9	136.30 (14)	C5—C6—H6	118.1
C8—Cu1—Br1^i^	104.22 (11)	S1—C7—H7A	108.5
C8—Cu1—Br1	141.26 (11)	S1—C7—H7B	108.5
C8—Cu1—C9	38.43 (15)	H7A—C7—H7B	107.5
C9—Cu1—Br1	104.57 (12)	C8—C7—S1	115.0 (3)
C9—Cu1—Br1^i^	107.05 (12)	C8—C7—H7A	108.5
C2—S1—C7	106.04 (17)	C8—C7—H7B	108.5
C2—N1—Cu1	128.0 (2)	Cu1—C8—H8	93.7
C2—N1—C6	117.2 (3)	C7—C8—Cu1	104.9 (2)
C6—N1—Cu1	114.5 (2)	C7—C8—H8	118.0
N1—C2—S1	122.9 (3)	C9—C8—Cu1	71.5 (2)
N1—C2—C3	122.3 (3)	C9—C8—C7	123.9 (4)
C3—C2—S1	114.7 (3)	C9—C8—H8	118.0
C2—C3—H3	120.3	Cu1—C9—H9A	104.7
C4—C3—C2	119.3 (4)	Cu1—C9—H9B	95.0
C4—C3—H3	120.3	C8—C9—Cu1	70.1 (2)
C3—C4—H4	120.4	C8—C9—H9A	120.0
C3—C4—C5	119.1 (4)	C8—C9—H9B	120.0
C5—C4—H4	120.4	H9A—C9—H9B	120.0
			
Cu1—N1—C2—S1	−6.5 (4)	C2—C3—C4—C5	1.2 (5)
Cu1—N1—C2—C3	171.5 (3)	C3—C4—C5—C6	−1.3 (6)
Cu1—N1—C6—C5	−172.5 (3)	C4—C5—C6—N1	0.1 (6)
S1—C2—C3—C4	178.3 (3)	C6—N1—C2—S1	−179.3 (3)
S1—C7—C8—Cu1	−74.2 (3)	C6—N1—C2—C3	−1.4 (5)
S1—C7—C8—C9	−151.7 (3)	C7—S1—C2—N1	−20.3 (3)
N1—C2—C3—C4	0.2 (5)	C7—S1—C2—C3	161.7 (3)
C2—S1—C7—C8	68.3 (3)	C7—C8—C9—Cu1	95.9 (3)
C2—N1—C6—C5	1.3 (5)		

Symmetry code: (i) −*x*+1, −*y*+1, −*z*+1.

### Di-µ-bromido-bis({2-[(η-2,3)-(prop-2-en-1-yl)sulfanyl]pyridine-κN}copper(I)) (II) Hydrogen-bond geometry (Å, º)

**Table:**

*D*—H···*A*	*D*—H	H···*A*	*D*···*A*	*D*—H···*A*
C3—H3···Br1^ii^	0.95	3.02	3.696 (4)	129
C6—H6···Br1	0.95	2.94	3.576 (4)	126
C7—H7*B*···Br1^iii^	0.99	2.94	3.744 (4)	139

Symmetry codes: (ii) *x*+1, *y*, *z*; (iii) −*x*+1, *y*+1/2, −*z*+1/2.
